# Operationalizing implementation science frameworks to plan a hybrid effectiveness-implementation study of a digital health intervention

**DOI:** 10.21203/rs.3.rs-5347341/v1

**Published:** 2024-11-20

**Authors:** Jacqueline Hodges, Wendy Cohn, Amanda D. Castel, Tabor Flickinger, Ava Lena D. Waldman, Michelle Hilgart, Olivia Kirby, Sylvia Caldwell, Karen Ingersoll

**Affiliations:** Duke University; University of Virginia; The George Washington University; University of Virginia; University of Virginia; University of Virginia; The George Washington University; University of Virginia; University of Virginia

**Keywords:** mobile health, digital health, HIV, RE-AIM, CFIR, hybrid effectiveness-implementation study, process, planning

## Abstract

**Background:**

Evaluating implementation of digital health interventions (DHIs) in practice settings is complex, involving diverse users and multistep processes. Proactive planning can ensure desired implementation determinants and outcomes are captured for hybrid studies, but operational guidance for DHI studies is limited.

**Methods:**

We planned a cluster randomized, type II hybrid effectiveness-implementation trial testing PositiveLinks, a smartphone application for HIV care, compared to usual care (n = 6 sites per arm), among HIV outpatient sites in the DC Cohort Longitudinal HIV Study in Washington, DC. Our process included: 1) Defining components of the DHI and associated implementation strategy, 2) Selecting implementation science frameworks to accomplish evaluation aims, 3) Mapping framework dimensions, domains, and constructs to implementation strategy steps, 4) Modifying/creating instruments to collect data for implementation outcome measures and determinants and 5) Developing a compatible implementation science data collection and management plan.

**Results:**

Specification of components of the DHI and implementation strategy identified relevant platform usage data and necessary implementer actions, toward planning measurement of program reach and adoption. *A priori* mapping of implementation strategy steps to the Reach Effectiveness Adoption Implementation Maintenance (RE-AIM) framework identified how data would be collected for each step/corresponding outcome measure. The multi-site cohort study provided infrastructure for prospective, scaled implementation research, including site research assistants (RAs) coordinating implementation. Existing cohort tools (periodic site assessments, patient consent logs) were adapted for the evaluation to further capture representativeness and reach/adoption ‘denominators.’ New survey instruments allowed for framework-guided evaluation of provider adaptations/dose/fidelity to planned implementation across a large number of participants and multiple timepoints. Some aspects of real-world implementation were challenging to mirror within the planned hybrid trial (e.g. RAs selected as de facto site implementation leads) or were modified to preserve internal validity of effectiveness measurement (e.g. PositiveLinks ‘community of practice’).

**Conclusions:**

Challenges and opportunities arose in planning the implementation evaluation for PositiveLinks within a hybrid trial in a cohort population. Prospective hybrid trial planning must balance generalizability of implementation processes to ‘real world’ conditions, with rigorous trial procedures to measure intervention effectiveness. Rapid, scalable tools require further study to enable evaluations within large, multi-site hybrid studies.

## Background

Digital health interventions (DHIs) including web-based and mobile health (mHealth) interventions can improve clinical outcomes for chronic health conditions. DHIs utilize behavior change theories with variable mechanisms of action, such as enhanced motivation, self-management, and peer support. DHIs may be embedded within, or replace, traditional care, and engage different end-users (e.g. patients, providers, or both). Munoz et al (1) and Hermes et al (2) describe the spectrum of behavioral intervention technologies ranging from adjunctive tools to support provider tasks or strengthen patient-provider communication, to fully-automated direct-to-consumer technologies for patient self-management. The range of stakeholders, roles, and inputs needed to implement DHIs can be complex, involving multi-level implementation processes, environments, and permissions. Additionally, DHIs generate backend metadata/paradata about patient and provider usage. These complex, interacting factors require a systematic, pragmatic, scaled implementation research approach to evaluate implementation outcomes and determinants. Curran et al. describe three archetypes of hybrid effectiveness-implementation studies (3), however the literature provides little operational guidance or shared procedural knowledge on effectively planning hybrid studies to capture implementation outcomes and identify salient implementation determinants for complex DHIs.

There is also a growing need within the field of DHI implementation research to evaluate implementation outcomes beyond user preferences and other outcomes (e.g. acceptability, usability, feasibility) typically reported in formative development studies (4, 5) and human-centered design (6–10). Under-studied implementation outcomes for DHIs include fidelity of implementation and adaptations made to DHIs and associated implementation strategies within varied practice settings (9, 11), as well as intervention penetration and sustainability which require pragmatic trial designs to reflect ‘real-world conditions’ (7, 9, 12). We reviewed several comprehensive efforts to re-characterize or adapt broader health service implementation science frameworks for the study of DHIs. Recent examples include 1) a workshop conducted by the Dissemination and Implementation Core of the Center for Technology and Behavioral Health at Dartmouth College (13), 2) Hermes et al.’s re-characterization of Proctor’s outcomes for implementation research for technology-based behavioral interventions (2), and 3) De la Vega et al.’s (14) post hoc application of this re-categorized framework against Glasgow’s Reach Effectiveness Adoption Implementation Maintenance (RE-AIM) framework (15), which itself has previously been applied in DHI implementation research (16). Determinant frameworks can elucidate barriers and facilitators of various DHIs, including the Consolidated Framework for Implementation Research (CFIR) (17–20), the Theoretical Domains Framework (21), Promoting Action on Research Implementation in Health Services (22), and others. Application of these frameworks is often performed post hoc only, and proactive processes to operationalize framework components within DHI implementation studies are not well defined in the literature.

The PositiveLinks platform is a clinic-deployed multi-feature smartphone application with patient and provider-facing components. It was developed and refined following a rigorous, iterative process of user-centered design to support people with HIV (PWH) receiving outpatient care (23). Program implementation among a cohort in Central Virginia where the intervention was developed, tested and refined has demonstrated long-term usage and significant improvement in clinical outcomes at 12 (24) and 24 months (25). The platform has been adapted for several other chronic conditions, end-users and contexts (26–31). To date, PositiveLinks has been implemented as part of routine clinical care at 9 clinics in Virginia, and 8 sites in other states, and is considered an evidence-based intervention for HIV care by several national consensus guidelines. Clinical effectiveness of PositiveLinks is currently being tested against usual care in a hybrid trial, the PositiveLinks in DC Cohort Study, using a cluster randomized controlled trial design (32). The trial is being conducted among sites in the DC Cohort Longitudinal HIV Study (DC Cohort Study) following over 12,800 PWH at 14 outpatient HIV practice settings, including Federally Qualified Health Centers and academic medical centers (33). We share our process to proactively define, prioritize, and operationalize measurement of relevant implementation outcomes for this type II hybrid effectiveness-implementation trial testing a complex DHI, and elucidate determinants of implementation among six DC Cohort sites randomized to the intervention over a 12-month study period. We highlight the unique opportunities and challenges that emerged for planning of a hybrid DHI trial among a large-scale epidemiologic cohort study population.

## Methods

### Study team and process refinement

The hybrid trial planning phase spanned an 18-month period preceding onboarding of the first site in December 2022, conducted by an interdisciplinary research team. Research team members hold an established record of clinical research experience, including conducting formative evaluations and observational studies testing clinical efficacy of PositiveLinks. Investigators’ primary expertise include: clinical psychology, program evaluation, qualitative methods, instructional design, software development, and implementation research. Program managers contributed empirical observation of PositiveLinks implementation processes over a decade that assisted with conceptualization of the intervention, implementation contexts, and components of the implementation strategy. The trial planning team also included DC Cohort Study investigators with expertise in epidemiologic and intervention studies at the cohort sites. The methodologic approach to proactively integrate implementation evaluation activities within the hybrid trial required iterative steps conducted through multiple cycles of team feedback, consensus, and refinement.

#### 1. Define components of the digital health intervention and associated implementation strategy

We conceptualized then specified components of the DHI implementation process and discrete steps of the implementation strategy specific to the context of the DC Cohort. Necessary strategy steps carried out by implementers have emerged over prior implementations where PositiveLinks is part of usual care. Key necessary in-clinic strategy steps, associated barriers, and facilitators to implementation were synthesized from previous implementations in a rapid evaluation study conducted for PositiveLinks implementation across several sites in Central Virginia (34). Implementation strategy steps were further specified in terms of actors, corresponding actions, and action targets within the DC Cohort Study infrastructure. Proctor et al. describe specifying complex implementation strategies (35) to facilitate appropriate measurement, and Fernandez et al. describe the process of selecting appropriate implementation strategies using implementation planning (36), including identification of performance objectives to describe ‘who has to do what.’ Our process contextualized the existing implementation strategy and corresponding performance objectives that could be studied for PositiveLinks implementation within the hybrid study conducted among the DC Cohort.

#### 2. Select appropriate implementation science frameworks to accomplish evaluation aims

We then identified aims for the implementation evaluation arm of the hybrid trial: Aim 1) define and measure implementation outcomes of interest and Aim 2) elucidate determinants of implementation in a rapid, scaled fashion across participating sites. We first used narrative reviews of theories, models, frameworks, and strategy categorization to assess the most widely used determinant and evaluation frameworks (37, 38). We then reviewed technology-specific compendia and original research studies re-conceptualizing broader health service frameworks toward DHIs, including hybrid study designs (2, 13, 14, 16, 18). We subsequently selected the RE-AIM evaluation framework and CFIR determinant framework for our first and second implementation evaluation aims, respectively.

#### 3. Map framework dimensions, domains, and/or constructs to steps of the implementation strategy

We mapped each dimension of the evaluation framework, RE-AIM, to specified components of the intervention and implementation strategy steps, prioritizing specific steps for evaluation based on impact for future implementations and feasibility of measurement during the hybrid trial study period.

We generated corresponding implementation outcome measures and identified corresponding methods of data collection: 1) **creation** of new instruments for prospective data collection specific to the implementation evaluation, 2) **modification** of standardized tools used for DC Cohort intervention studies or PositiveLinks evaluations or 3) **abstraction** from existing PositiveLinks or DC Cohort sources (e.g. DC Cohort Database storing patient encounter, laboratory, and sociodemographic data).

We selected salient domains/constructs identified from the prior qualitative CFIR-guided assessment of several sites implementing PositiveLinks (34). We planned to convert salient CFIR interview guide questions into survey items to be rapidly deployed among all provider participants at multiple pre-defined timepoints (baseline, 6, 12 months) in order to assess implementation determinants in a scaled, rapid fashion. As approximately 50 or more providers could be approached for enrollment across trial intervention sites, we planned CFIR-guided qualitative interviews to elicit open-ended feedback from a limited subset of providers post-implementation. Similarly, post-implementation patient focus groups were planned for a subset of patients given the scaled nature of the study.

#### 4. Modify or create instruments to support data collection for implementation outcome measures and determinants measures and determinants

Data collection instruments were modified with additional items or created *de novo* within Research Electronic Data Capture (REDCap) to completely assess each RE-AIM dimension and corresponding implementation outcome measures. For RE-AIM dimensions and CFIR constructs/domains of interest typically probed using open-ended survey items or qualitative interview questions, respectively, corresponding provider survey items were generated using close-ended questions (e.g., dichotomous or Likert scale responses), while allowing providers to elaborate as needed with optional free-text responses.

#### 5. Develop a compatible implementation science data collection and management plan

Finally, we generated an overall plan for participant data collection and management that ensured compatibility between the clinical effectiveness arm of the trial and the implementation evaluation. Plans for data collection and abstraction were incorporated into patient approaches for follow-up and study monitoring already planned for the trial’s effectiveness arm.

## Results

### Define components of intervention and implementation strategy

**Figure 1:**
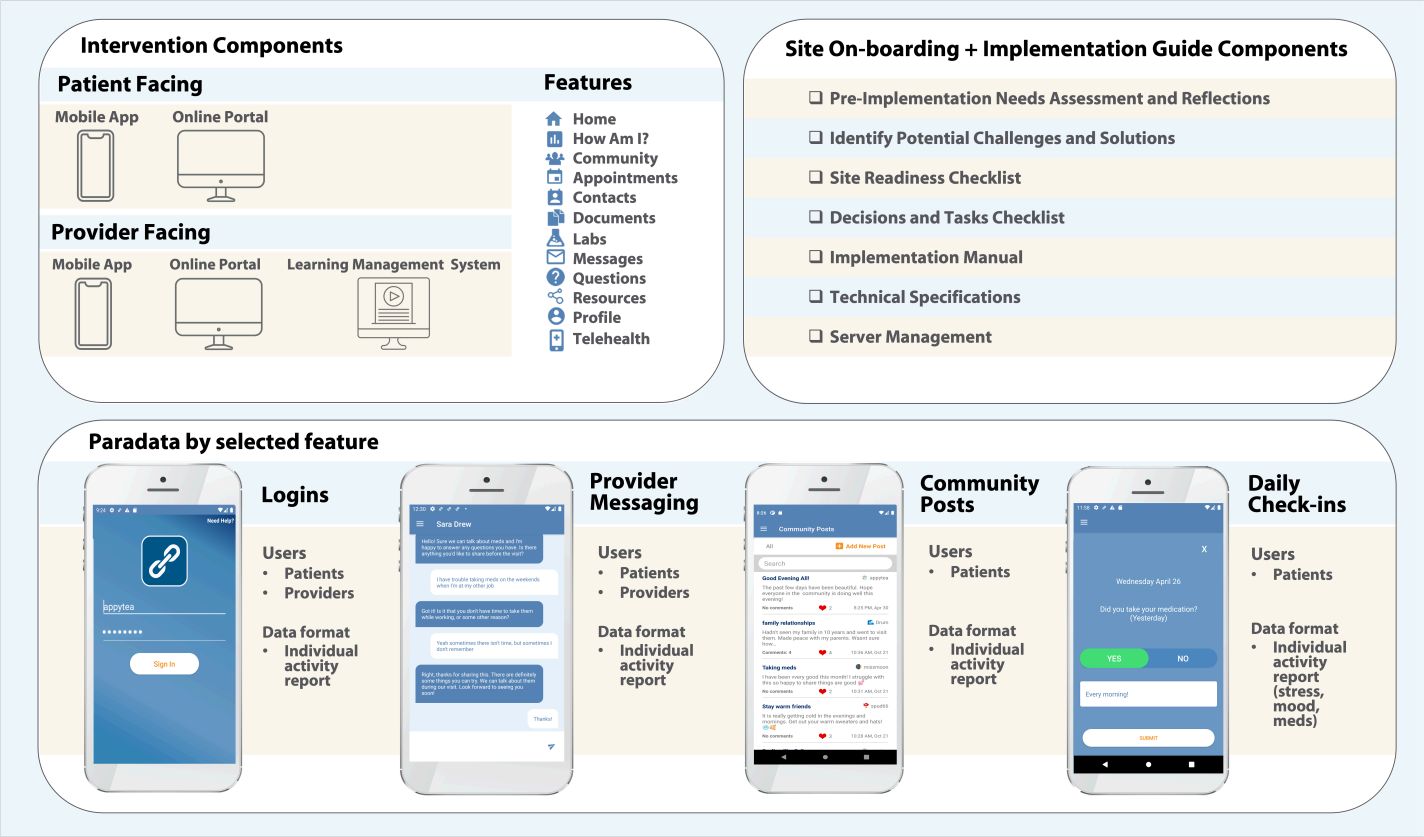
Specified components of PositiveLinks intervention, site on-boarding/implementation support materials and platform paradata.

Step 1 of the planning phase included specification of PositiveLinks intervention components (both patient and provider-facing elements) for initial site on-boarding and longitudinal implementation, and paradata for usage of each feature (Fig. 1).

Implementation guide documents refined over the course of ‘real-world’ PositiveLinks implementations focus on generating group discussions and prompting action items for providers (site leadership, physicians, case managers, social workers, clinical nurses, and other support staff) to prepare for implementation. Intervention components and guides were further iterated based on feedback obtained from Cohort stakeholders during the formative evaluation conducted to tailor PositiveLinks for the DC Cohort context (39). Backend paradata are available via the platform and were specified by user group and data format (available individual activity reports including feature usage frequency over selected time intervals, or free-text post content) in order to determine desired metrics of uptake for study implementation outcomes defined in steps 2 and 3.

**Figure 2:**
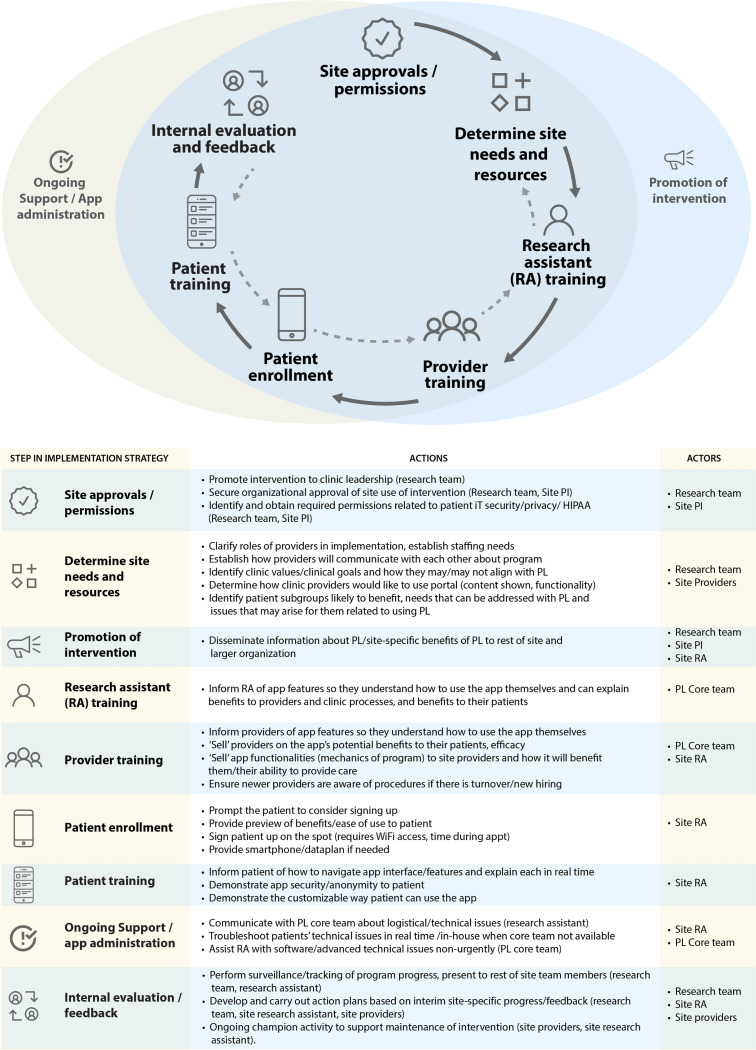
Discrete implementation strategy steps expected for program deployment at DC Cohort sites. RA=research assistant, PI=principal investigator.

Steps of the planned implementation strategy specified for the DC Cohort context are shown in Fig. 2. ‘Actors,’ include the PositiveLinks core team’s program managers who assist remotely with implementation activities and troubleshooting at partner sites. The study team includes researchers involved with coordinating the specific intervention study being conducted to test PositiveLinks in the DC Cohort.

Site on-boarding for PositiveLinks implementation conducted by the PositiveLinks core team spans multiple outlined steps of the implementation strategy expected for the DC Cohort (determine site needs and resources, conduct research assistant (RA) and provider training). Study team members have a larger role in these strategy steps for the hybrid trial. Additional distinctions for planned implementation among DC Cohort sites within the hybrid trial emerged as compared to real-world implementation. First, Cohort sites agreed to participation in the trial, each with a DC Cohort Study principal investigator overseeing trial activities at respective sites. RAs employed at DC Cohort Study sites are familiar with recruiting and consenting patients, uploading relevant patient data, and conducting assessments throughout the study period. Site RAs were identified as actors to conduct several steps of the implementation strategy in this context, whereas in real-world implementation, the program is administered by individuals with a range of roles, typically identified by leadership or self-identified as the site lead for implementation. Second, real-world PositiveLinks implementation offers collaboration between sites in a ‘community of practice,’ to share implementation challenges, solutions and unique adaptations to improve patient experiences and engagement. In contrast, we sought to maintain internal validity and avoid cross-site contamination for the trial, so intervention sites were not included in the community of practice. Third, steps such as administration of technical app support and troubleshooting as-needed and promotion of the intervention are usually more informally evaluated through site discussions and internal meetings, and were planned to be evaluated only in qualitative study activities.

### Select appropriate implementation science frameworks to accomplish evaluation aims and map framework dimensions, domains and constructs to steps of the implementation strategy

**Figure 3:**
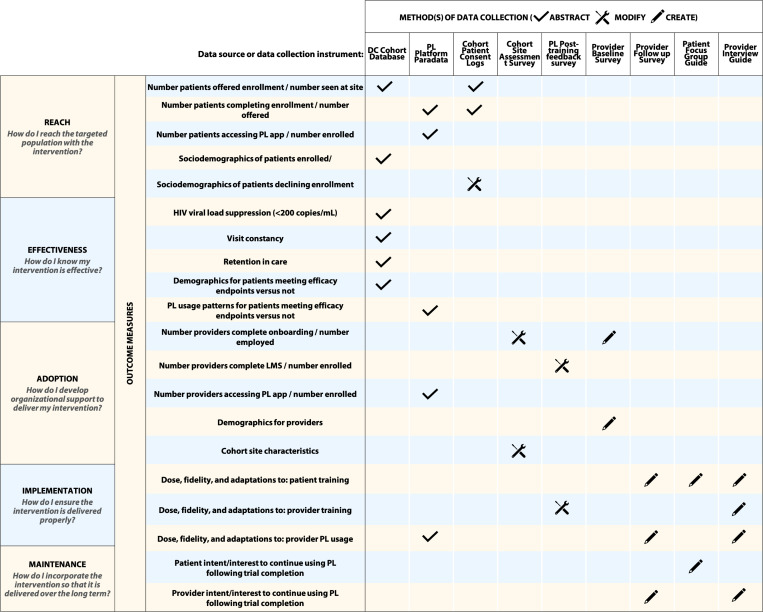
RE-AIM Framework mapped to intervention/implementation strategy, corresponding outcome measures, data collection methods. Data collection methods include abstraction from one or more items from existing data sources, modification or addition of items to existing data collection instruments, or creation of new instruments. PL = PositiveLinks, LMS = learning management system. Visit constancy is the proportion of 4-month intervals a visit is completed in 12 months. Retention in care per HRSA-1 definition: 2 appointments attended at least 90 days apart within 12 months.

Output for steps 2 and 3 are summarized in Fig. 3. Specifically, RE-AIM evaluation framework dimensions were mapped to each prioritized step within the implementation strategy. Corresponding methods of data collection were identified for each resulting outcome measure. Distinctions between real-world implementation processes and those expected for the trial also emerged at this stage. The learning management system (LMS) is a series of interactive training modules developed for providers and staff, aimed primarily at increasing knowledge and familiarity with the use of platform features (followed by a Post-training Feedback Survey). Prior to site on-boarding for the study, several Cohort site PIs expressed they would expect significantly lower enrollment of providers if LMS completion was mandatory, primarily due to significant provider clinical burden and/or competing demands. As a result, participating providers could opt out of LMS completion, but were expected to undergo group on-boarding or one-on-one training with the site RA prior to initiating program participation.

### Modify or create instruments to support data collection for implementation outcome measures

Several modifications to existing data collection instruments for DC Cohort intervention studies were required. The DC Cohort Site Assessment Survey is periodically deployed to Cohort sites for updated assessment of available service delivery. Several existing assessment items were identified for abstraction for evaluation of the adoption dimension of RE-AIM (e.g., on-site clinical services and support services, updated staffing of clinical and non-clinical providers, specialty training). We added several items to capture the denominator of people employed for each type of provider role (e.g., attending physician, clinic nurse, case manager, social worker, etc) as well as site-level baseline mHealth/technology use (Additional file 1) to more adequately assess the adoption dimension for PositiveLinks implementation. Patient consent logs standardized for the DC Cohort Study include patients’ reasons for declining participation, with up to three approaches. These logs were modified to query a selected number of demographics for Cohort participants declining to participate in the study (age, sex, race, ethnicity, insurance status, last CD4 count and last HIV viral load), allowing for evaluation of representativeness of non-participants (Reach dimension).

Instruments created specifically for the implementation arm of the hybrid trial included surveys directed at providers not usually targeted by data collection for intervention studies at Cohort sites. The provider baseline survey (Additional file 2) and follow up survey (Additional file 3), annotated with respective framework components) were designed to capture otherwise un-incorporated datapoints for measurement for the implementation dimension of RE-AIM (e.g. fidelity, dose and any adaptations made to steps of the implementation strategy by site providers or RAs). We incorporated the Organizational Readiness to Implement Change (ORIC) tool into the provider baseline survey to assess collective baseline readiness at each site (40, 41). Salient implementation determinants adapted for inclusion from our prior CFIR-guided rapid evaluation study of PositiveLinks real-world implementation were: Inner Setting (Compatibility), Outer Setting (Patient Needs and Resources, External Policy and Incentives), Characteristics of Individuals (Knowledge and Beliefs), Innovation Characteristics (Adaptability, Complexity), and Implementation Process (Planning, Engagement of Key Stakeholders). Post-implementation patient focus group and provider in-depth interview guides were also adapted from prior PositiveLinks implementations using salient CFIR 1.0 domains/constructs.

### Develop a compatible implementation science data collection and management plan

Finally, we developed a plan to specify timing and frequency of data abstraction (e.g. from the DC Cohort Database) and collection in relation to planned activities for the effectiveness arm of the trial (e.g. patient consent/enrollment, administration of baseline, 6-month, and 12-month assessments). For patient data, plans were designed to minimize separate approaches as well as ‘data pulls’ from existing sources anticipated to support evaluation of clinical effectiveness outcomes. Further, monitoring of feature usage from platform paradata for patients and providers is a routine part of real-world PositiveLinks implementation, to guide efforts to engage and re-engage staff and enrolled patients, troubleshoot concerns in real-time, and ensure sustainability. Frequency of paradata abstraction for monitoring was thus pre-determined for specific features at timepoints throughout implementation. We required informed consent for all provider activities in the evaluation, including collection of provider survey responses and participation in post-implementation in-depth interviews. Selection of the timing and frequency of provider survey administration required consideration of provider turnover, particularly in participating intervention sites with higher expected turnover (e.g., trainees like infectious disease fellows rotating within academic centers).

## Discussion

We describe a team’s experience planning and proactively integrating implementation science methodology into a hybrid DHI study conducted within an existing cohort study. To apply relevant implementation science frameworks to DHI implementation evaluation, we reviewed how investigators compared Proctor’s Outcomes for Implementation Research recategorized for DHIs against RE-AIM (2, 14). These and other original research studies had limitations including a lack of measurable eligible ‘denominators’ available for certain outcomes (e.g. provider adoption dimension of RE-AIM). Prior studies also focused on conceptualizing outcomes for a primarily patient-facing intervention itself (e.g. different ways of leveraging back-end usage capture data and/or tracking referrals of patients by clinic to the study), rather than including additional strategy steps to implement a dual-facing (patient and provider) intervention (mapped in Fig. 2), which each require dedicated measurement of provider inputs as well.

While RE-AIM offered adequate operational guidance to evaluate this DHI and implementation strategy, several lessons, opportunities, and challenges arose throughout the planning process. There is a well-recognized tension between maintaining the rigor and validity of randomized trial designs and establishing pragmatic conditions more relevant to real-world implementation (42). By performing a pre-implementation planning phase for the trial, incorporating frameworks proactively, we ensured collection of a robust set of quantitative and qualitative data. In contrast, in post hoc evaluations performed in ‘real world’ conditions expected of implementation research as most strictly defined, there are limits on the extent to which additional implementation outcomes like site-specific fidelity, dose, program adaptations, and uptake of specific components or steps of implementation can be evaluated with sufficient granularity and coverage of participants over the course of implementation. Simultaneously, however, the tighter control of prospective hybrid trial conditions and administration of the program through protocolized research activities inherently limits how well ‘real-world’ conditions are reflected within a hybrid trial.

Real-world PositiveLinks implementation often relies on outreach by partner sites, by individuals who serve as champions of the intervention with an active role in obtaining site approvals (related to data security, patient privacy, etc.), and who continue to promote the intervention. RAs assigned as de facto program managers at sites participating in the hybrid trial, however, is a major distinction from real-world implementation. This distinction, while needed to rapidly plan and conduct a multi-site trial, challenges generalizability of prospective DHI implementation research when the interventions require a distinct, multi-layered ‘implementation climate.’ These ‘climates’ typically require gradual building of multiple, interacting implementers’ self-efficacy, motivation, and longitudinal intervention promotion efforts to ensure site readiness and penetration.

Additional considerations for hybrid trials implementing DHIs may represent challenges to generalizability in terms of real-world maintenance and sustainability, including providing technology to patients (e.g., smartphones, data plans), incentives for usage of the intervention and/or specific features, and participant retention protocols common for clinical efficacy trials. Real-world PositiveLinks implementation variably includes provision of phones and/or data plans, depending on specific partner site funding availability and patient need, and re-engagement protocols are also a routine part of implementation at several sites. For this trial, we planned to rely on existing site-specific or federal subsidy programs available to participants in the context (e.g., Federal Communications Commission Lifeline program) before providing smartphones and/or data plans, and a retention protocol was used by site RAs to periodically re-engage patients. No additional incentives were planned for higher levels of app usage, however, and patients who do receive smartphones can keep them for the duration of the study regardless of app usage.

Examining implementation in parallel with a cluster randomized effectiveness trial among an epidemiological cohort presents yet another set of challenges and opportunities. This hybrid approach allows for a scaled implementation evaluation to occur across multiple sites simultaneously, leveraging existing research infrastructure, including site staffing with RAs and existing data collection instruments. For our study, the DC Cohort patients who chose not to participate in the PositiveLinks program at their respective sites could be examined for sociodemographic representativeness, a component of patient ‘reach’ within RE-AIM that appears infrequently in published applications of this framework. The scaled, simultaneous multi-site approach, however, engages a larger number of patients and providers within time and funding constraints of a single hybrid trial, and necessitates consideration of more rapid, cost-effective approaches to implementation evaluation (acknowledged by de la Vega et al) (14). Provider surveys, for example, were designed to capture implementation outcome measures and determinants for the larger expected sample of respondents at six intervention sites than are typically engaged with more in-depth qualitative processes applying these frameworks, in particular CFIR. There is not consensus in the implementation science field about how these frameworks reflecting complex psychosocial/behavioral constructs should be applied, including whether to attempt to dichotomize or categorize items for broader, rapid distribution. These efforts could, for example, introduce study team bias and reduce the systematic nature of framework application (38). Combined in-depth and more streamlined approaches can offer opportunities to validate the latter methods, but more extensive psychometric validation of tailored surveys applying these frameworks for complex DHIs are needed to ensure generalizability and validity.

## Conclusions

Implementation research for complex DHIs can expand understanding of how these interventions will behave in ‘real world’ conditions. Prospective hybrid effectiveness-implementation trials can facilitate more in-depth implementation evaluations at scale if appropriately planned. Our experience highlights the ways in which evaluations must attempt to balance rigor, proximity to ‘real world’ implementation climates, and incorporate multiple key implementation outcomes and determinants within the time and resource constraints of a prospective DHI hybrid trial. Generated data collection instruments targeting provider-related outcomes and determinants for DHI implementation require further validation.

## Supplementary Material

Supplement 1

## Data Availability

The datasets used and/or analyzed during the current study are available from the DC Cohort Longitudinal HIV Study following approval of request(s) from the study team and DC Cohort Executive Committee.
