# Supplementary material for: Operationalizing implementation science frameworks to plan a hybrid effectiveness-implementation study of a digital health intervention
Source: Res Sq. 2024 Nov 20:rs.3.rs-5347341. Preprint. [Version 1] doi: 10.21203/rs.3.rs-5347341/v1 (PMC11601874; doi:10.21203/rs.3.rs-5347341/v1)
Supplement: Supplement 1 [file NIHPPRS5347341V1-supplement-1.pdf]

Supplementary Files

This is a list of supplementary files associated with this preprint. Click to download.

- AdditionalFile1DCCohortSiteAssessmentSurveyModifications.docx
- AdditionalFile2ProviderBaselineSurveys.docx
- AdditionalFile3ProviderFollowUpSurveys.docx
- AdditionalFile4CONSORTEHEALTHV1.6.1Checklist.pdf
